# What to target in cognitive behavioral treatment for gambling disorder—A qualitative study of clinically relevant behaviors

**DOI:** 10.1186/s12888-022-04152-2

**Published:** 2022-07-28

**Authors:** Olof Molander, Jonas Ramnerö, Johan Bjureberg, Anne H. Berman

**Affiliations:** 1 Department of Clinical Neuroscience, Center for Psychiatry Research, Karolinska Institutet, Norra stationsgatan 69, plan 7, 113 64 Stockholm, Sweden; 2 Stockholm Region Health Services, Stockholm, Sweden; 3 Department of Psychology, Uppsala University, Uppsala, Sweden

**Keywords:** Gambling disorder, Functional assessment, Cognitive behavioral treatment

## Abstract

**Background:**

From a clinical perspective, knowledge of the psychological processes involved in maintaining gambling disorder has been lacking. This qualitative study formulated hypotheses on how gambling disorder is maintained by identifying clinically relevant behaviors at an individual level, as a means to guide the development of new cognitive behavioral interventions.

**Methods:**

Six individuals from a treatment study, diagnosed with gambling disorder and with diverse symptom profiles of psychiatric comorbidity, were recruited. Participants were interviewed using an in-depth semi-structured functional interview and completed self-report measures assessing gambling behavior.

**Results:**

Functional analysis was used as a theoretical framework for a thematic analysis, which yielded the following categories: 1) antecedents that may increase or decrease gambling; 2) experiences accompanying gambling; 3) control strategies; 4) consequences of gambling behavior; and 5) events terminating gambling behavior. Few differences were identified in relation to symptom profiles of psychiatric comorbidity, although some gamblers did not report experiencing abstinence when not being able to gamble.

**Conclusions:**

Gambling is a secluded activity mainly triggered by access to money. Positive and negative emotions could be both antecedents and functions of gambling behavior. Avoidance-based strategies used to control gambling might result in a failure to learn to control gambling behavior. Anticipation, selective attention, and chasing could be important reinforcers, which should be addressed in new developments in cognitive behavioral treatment for gambling disorder.

**Supplementary Information:**

The online version contains supplementary material available at 10.1186/s12888-022-04152-2.

## Introduction

Gambling, an activity “where something of value is risked on the outcome of an event when the probability of winning or losing is less than certain” [1], is a behavior that has generated increased interest in research and clinical practice. Gambling has been called a “pure” addiction from a behavioral perspective [2], in that it lacks any form of involvement from external chemical agents, and it was the first such state acknowledged as an addiction disorder. With the introduction of the 5th edition of the Diagnostic and Statistical Manual of Mental Disorders (DSM-5; [3]), gambling was equated with substance use as an addiction. *Gambling disorder* (GD, previously called pathological gambling), includes behaviorally-based criteria such as loss of control, chasing losses, increased tolerance and gambling as an escape from aversive experiences. The past year prevalence of *problem gambling*, meaning gambling leading to any negative consequences, varies across countries between 0.3% and 5.3% in the general population [4]. Both problem gambling and GD are associated with severe negative consequences in important life domains such as finances, wellbeing, relationships and poorer mental and physical health, including higher rates of suicide ideation and attempts, for both the person with gambling problems and their significant others [5–7]. Furthermore, problem gambling and GD are highly comorbid with other psychiatric disorders [8, 9]. Taken together, these recent developments indicate that increasing understanding of gambling as a behavior is a pivotal task, through basic research that can inform treatment development.

The phenomenon of learning and maintenance of unhealthy gambling habits has elicited a variety of attempts at explanation. It has been argued that gambling behavior has an intuitive fit to learning theories, in that gambling involves behavior under close control of rewards [10]. The phenomenon of gambling has been studied experimentally, with the investigation of several behavioral processes such as delay and probability discounting [11–13], reinforcement without actual winning [14–16], and rule-governed behavior [17]. Models of distinct gambling-related vulnerabilities have also been proposed. The Pathways model [18], suggests three subtypes manifesting impaired control over problematic gambling behavior: (1) Behaviorally conditioned gamblers who gamble due to learning processes such as conditioning and habituation; (2) emotionally vulnerable gamblers who gamble in order to relieve aversive experiences; and (3) impulsive/antisocial gamblers who gamble due to impulsive traits, substance use and antisocial behavioral tendencies. The Pathways model has gained increased prominence in the gambling field (e.g., [19–22]), but research has not shown whether the subtypes manifest different clinically relevant behaviors.

Treatment research on GD is a field still in its infancy. Currently, the only evidence-based treatments for GD are cognitive behavioral treatments (CBT). Clinical trials have shown CBT to be effective for reducing gambling behavior and related problems, but have failed to demonstrate differences between various treatment approaches (e.g., cognitive therapy, behavioral therapy, CBT and motivational interviewing), as well as between treatment and active control conditions [23–26]. Most current CBT approaches for gambling comprise a broad mixture of general CBT interventions found effective for other psychiatric conditions (for a review of treatment components, see [27]), but lack a solid theoretical base concerning the critical question of why gambling may persist despite obvious negative consequences [28].

Learning theory has served as a key inspiration for developing many psychological treatment models. However, behavioral treatment models and interventions for gambling have a less clear relation to basic experimental studies. On the other hand, experimental studies rarely involve clinical subjects or the natural environmental contingencies where the problematic gambling behavior occurs [29]. Behavioral principles are generally studied under strict observation and experimental control. In the prototypical clinical situation, i.e., talk therapy, the behaviors at hand are verbal descriptions of behavior given by the client, which may seem like a detour from a learning perspective. However, it could be argued that these narratives, in themselves, deserve attention as important data. Or as formulated by Foxall [30]:

*“The testimony that people give us about their intentions, plans, hopes, worries, thoughts and feelings is by far the most important source of information we have about them” (p. 112).*

A few existing qualitative studies were identified that examined gamblers’ subjective experiences in relation to their gambling, In her book, *Addiction by design* [31], the anthropologist Natasha Schüll interviewed members of Gambling Anonymous about their experiences of gambling. One striking feature in these subjective testimonies is that the role of winning money is downplayed as a motivating factor. Instead, a trancelike state that occurs with repetitive gambling, referred to as “the zone”, is more central. The zone is a state where the daily worries and concerns fade away in an almost dissociative manner. An interview study with a specific group of gamblers with schizophrenia found that they gambled specifically as a means to engage in a social activity, but also that their psychotic symptoms led to greater involvement in their gambling experience [32]. Finally, Hodgins & El‐Guebaly [33], interviewed recovered gamblers and found that they reported mainly emotional and financial reasons for quitting. Furthermore, the most endorsed actions taken in order to quit gambling were stimulus-control strategies, i.e., limiting access to gambling by avoiding gambling milieus or restricting access to money, and engaging in new, alternative activities. Starting with interviewing the afflicted is a clinically sensible strategy [34, 35], but it is also in line with the American Psychological Association (APA) presidential task force on evidence-based practice in psychology [36] that advocated the use of multiple types of research evidence that can contribute to effective psychological practice, ranging from clinical observation and qualitative research to broad-scale randomized controlled trials.

In the present study we interviewed individuals with GD regarding their own perceptions of the functions of their gambling behavior, as part of our aim to develop a treatment model based on functional analysis of gambling behavior [28]. The gambling subtypes from the Pathways Model [18] were used to ensure a diverse sample of participants with GD. Self-report measures and a functional assessment interview were used to identify clinically relevant behaviors and formulate hypotheses concerning the maintenance of GD on an individual level, as a preparatory step to guide clinical interventions.

## Methods

### Participants

Theory-based clinical sampling was used in this qualitative exploratory study. Treatment seeking individuals with GD and other psychiatric comorbidities were purposively selected by a clinical psychologist from a separate CBT study at the Stockholm Center for Dependency Disorders [37], as representative for the gambling types delineated in the Pathways Model [18]. Participants were recruited to this study after completing all treatment sessions. To be included in our study the participant had to: (1) be identified as one of the gambling subtypes according to the Gambling Pathway Questionnaire (GPQ; [38]), (b) show a total score of >= 3 on the Problem Gambling Severity Index (PGSI; [39]), (c) be 18-85 years old, (d) read and write Swedish. Prior to inclusion in the original treatment study, participants were screened and assessed for GD and psychiatric comorbidity with the Structured Clinical Interview for Gambling Disorder (SCI-GD; [40]) and the Mini International Neuropsychiatric Interview version 7 (MINI-7; [41]). Although GD was not originally defined as an inclusion criterion, all participants in the study fulfilled GD diagnostic criteria. After six participant interviews, the representative clinical material was deemed sufficient, and no further interviews were conducted. No participants dropped out. Table 1 shows an overview of participant characteristics.

**Table 1:** Participant characteristics

Participant characteristics	
Age, years M (Sd)	34 (9)
Gender	*n* 4/6 men
Occupation	Working (*n* 4/6), sick or parental leave
Civil status	Single, married or in relationship (*n* 2/6 own children)
Gambling types^a^	Casino online (*n* 4/6), casino on-site venue, sports-betting online, poker online, daytrading
Age first gambled, years M (Sd)	18 (3)
Duration of gambling problems, years M (Sd)	7 (6)
Number of GD criteria M (Sd)	7 (2)
Type value GD severity	Severe
Current psychiatric comorbidities	Alcohol use disorder, antisocial personality disorder, attention deficit hyperactivity disorder, depression, emotionally unstable personality disorder, generalized anxiety disorder, panic disorder, social anxiety disorder, suicide risk

*Note:* Patient characteristics were collected during baseline in the original treatment study [37]

^a^ The participants were able to report multiple gambling types

*GD* Gambling Disorder, *DSM-5* the Diagnostic and Statistical Manual of Mental Disorders 5^th^ edition

### Measures

The Functional Assessment Interview [42] is an open-ended semi-structured assessment instrument for clinical behavior analysis. For the purpose of this study the Functional Assessment Interview was adapted for gambling (FAI-G). To test the feasibility of the adapted interview, it was first piloted with one participant (not included in the study). After the pilot interview, the interview was modified further and shortened to focus on key features of gambling behavior. The final FAI-G interview (see Supplementary material 1) consisted of the following sections: Topography of gambling behavior, Antecedents of gambling behavior, Experiences when not being able to gamble, Physiological responses, Strategies to control and/or continue gambling, Experiences during gambling and function of gambling behavior, Terminating events of gambling behavior, and Behaviors with similar functions to gambling.

Self-report measures consisted of the Gambling Pathways Questionnaire (GPQ; [38], a 48-item self-report measure for assessing etiological gambling types according to the Pathways Model [18]; the revised version of Gambling Functional Assessment (GFA-R; [43]), a 16-item self-report measure developed to assess whether gambling behavior is maintained by positive reinforcement or escape; and lastly, the Centre for Addiction and Mental Health Inventory of Gambling Situations (CAMH-IGS; [44]), a 63-item self-report measure developed to assess high risk situations in the last 12 months that may have led to gambling behavior.

### Procedure

Participants were recruited from a separate treatment study. After completing an informed consent form and self-report measures online, they were contacted to arrange a face-to face FAI-G interview at a location of their own choosing. Three interviews were conducted at the Stockholm Center for Dependency Disorders clinic, two at the Center for Psychiatry Research, and one in the participant’s home. All FAI-G interviews except the pilot interview (not included) were carried out by the author OM, who took field notes. The interviews were audio recorded and subsequently transcribed and pseudonymized using a study id number. The interviews lasted between 60 and 90 minutes. Participants were given two movie vouchers as compensation.

### Data analysis

#### Qualitative analysis

Functional analysis as a theoretical framework (e.g., [45]) was used to review and analyze the transcribed FAI-G interviews. In the first step, two raters (authors OM and JB), independently reviewed and coded each FAI-G section in the transcribed interviews into short sentences or phrases. The raters, blinded to GPQ scores, also made independent clinical assessments regarding gambling type according to the Pathways Model [18], based on each participants FAI-G responses. In the next phase, the coded sentences and phrases were condensed further and coded into single words or short phrases. Any sentence or phrase bearing individual meaning and coded by either of the raters was added into a data pool, which also included information on the FAI-G section and participant id number. After this, each coded word or short phrase in the data pool was reviewed and categorized using theoretical thematic analysis [46]. Functional analytic themes were chosen that best described the most important concepts highlighted by the participants under each FAI-G section. The categorization and interpretation were done by authors OM and JR. Frequencies of endorsed constructs and phrases were summarized for all participants, as well as for each clinically assessed Pathways subtype [18]. In the last step, the results were triangulated among all authors. Results were reported in alignment with the Consolidated Criteria for Reporting Qualitative Research (COREQ) 32-item checklist [47].

#### Researchers’ competence

The researchers had complementary competences within different disciplines of clinical psychology. Author OM is a clinical psychologist, PhD, and researcher with experience of CBT development. Author JR is a clinical psychologist, PhD, and associate professor, with expertise in behavioral analysis and CBT. Author JB is a clinical psychologist, PhD, and researcher with experience of emotion regulation and CBT development. Author AHB is a clinical psychologist, PhD, and professor, with expertise in addiction. Variation in coding between raters were highlighted and discussed in detail, safeguarding that all perspectives were vocalized before consensus was reached. We are satisfied that this process ensured credibility and trustworthiness in interpretation and analysis.

#### Quantitative analysis

Descriptive statistics were used to present participant characteristics. Measure scores (GPQ, GFA-R, CAMH-IGS) were calculated for individual participants, as well as means and standard deviations for clinically assessed Pathways subtypes. Unweighted Cohen's κ for two raters [48] was used to calculate inter-rater reliability regarding Pathways subtypes by assessor 1 (first author) and assessor 2 (third author), as well as between clinical assessments and GPQ score. Quantitative analyses were performed using R Studio version 1.1.456 [49].

## Results

The mean participant age was 34 years (Sd=9.12), with 2/6 women. Casino online was the most frequent gambling type, played by 4/6. On average, participants reported onset of gambling problems 6 years and 7 months prior to inclusion. The mean number of fulfilled GD diagnostic criteria was 7 (Sd=1.72). Participants had an average of 1.7 additional DSM-5 psychiatric diagnoses, where anxiety disorders were most common. See Table 1 for individual participant characteristics, and Table 2 for self-report measures assessing gambling behavior.

**Table 2:** Results of self-report measures assessing gambling behavior

	Total (*N* = 6)	Pathway subtypes^a^		
		Conditioned (*n* = 2)	Emotional (*n* = 2)	Impulsive (*n* = 2)
GFA-R	M (Sd)	M (Sd)	M (Sd)	M (Sd)
Positive Reinforcement	19 (7)	18 (2)	26 (6)	13 (1)
Negative Reinforcement	25 (10)	17 (10)	33 (0)	26 (11)
CAMH-IGS^b^				
Negative Affect Situations				
Negative Emotions	66^c^ (16)	65^c^ (7)	65^c^ (3)	68^c^ (35)
Conflict with Others	46 (16)	57 (0)	34 (6)	48 (27)
Positive Affect Situations				
Pleasant Emotions	48 (20)	56 (33)	50 (14)	37 (14)
Social Pressure	27 (17)	12 (3)	36 (23)	34 (13)
Temptation Situations				
Urges and Temptations	74^c^ (13)	78^c^ (16)	70^c^ (16)	74^c^ (16)
Testing Personal Control	58 (23)	78^b^ (30)	48 (13)	48 (13)
Gambling Cycle Situations				
Need for Excitement	53 (19)	47 (35)	58 (4)	53 (20)
Worried about Debts	48 (21)	56 (33)	47 (28)	40 (10)
Winning and Chasing	48 (21)	84^c^ (23)	75^c^ (4)	70^c^ (28)
Confidence in Skill	56 (30)	54 (66)	54 (9)	60 (10)

Conditioned = Pathway 1 behaviorally conditioned subtype [18]

Emotional = Pathway 2 emotionally vulnerable subtype [18]

Impulsive = Pathway 3 antisocial impulsive risk-taking subtype [18]

*GFA-R* the Gambling Functional Assessment Revised, *CAMH-IGS* the Centre for Addiction and Mental Health Inventory of Gambling Situations [44]

^a^ Gambling Pathway subtypes [18] according to clinical assessment in this study

^b^ Results presented in Problem Index Scores

^c^ Clinical cut-off score (> 60) of the Centre for Addiction and Mental Health Inventory

of Gambling Situations

### Gambling Pathways subtype assessments

Two participants were clinically assessed as conditioned (Behaviorally Conditioned Subtype 1), two as emotional (Emotionally Vulnerable Subtype 2) and two as impulsive (Antisocial, Impulsive Risk-taking Subtype 3). Compared to clinical assessment, GPQ differently identified one conditioned participant as impulsive, one emotional as conditioned, and one emotional as conditioned. Perfect agreement was achieved for clinical assessments of Pathway gambling subtype between assessors 1 and 2, κ = 1, z = 3.46, *p* = 0.000. Agreement between clinical assessments and GPQ result was fair, κ = 0.25, z = 0.866, *p* = 0.386. The clinician-assessed Pathway subtypes were used for analysis of the results.

### Self-reported gambling behavior

Self-report measures indicated that gambling as a function of negative reinforcement was more common among clinically assessed emotional and impulsive gamblers, compared to conditioned gamblers. Similar results were found for positive reinforcement, but not for impulsive gamblers (see Table 2). In a similar vein, three antecedent high-risk situations for gambling behavior were above the clinical CAMH-IGS cut-off score, irrespective of clinically assessed gambling Pathway subtype: Negative emotions, urges and temptations, and winning and chasing.

### Functional assessment of gambling behavior

Coding of participant FAI-G interviews yielded 330 phrases, of which 258 were unique. Eight phrases were categorized as “Other: Not categorizable”, and were excluded from analysis. The thematic analysis yielded 23 functional analytic themes, within the FAI-G sections. See Table 3 for examples of coding and categorization, and Table 4 for frequency of these functional themes, as coded in the participants’ interviews.

**Table 3:** Examples of FAI-G participant answers, coding and categorization

FAI-G section	Participant answers	Coded short phrases	Functional themes
Antecedents that increase gambling behavior	“Mmm, today it will go well […] the race in on, great, let's go […] Sort of a feeling of exhilaration and expectation” Participant 6	“Exhilaration”, “Positive expectation” (assessor 1) “Exhilarated expectation” (assessor 2)	Emotion (positive or negative)
Strategies to control gambling	“I have tried to let my mother handle my economy, but we argue all the time and it drains our relationship” Participant 3	“Let mom handle the economy” (assessor 1) “Mother handles the economy “ (assessor 2)	Avoidance
Function and maintenance of gambling behavior	“I have gambled for the sake of gambling […]Everything else is pushed aside. The only thing that exists is focus on the game. An excitement, an anticipation, and yes, a joy […] Entering a sort of bubble” Participant 1	“Gambling for the sake of gambling”, “All focus on gambling, no other thoughts”, “Entering a bubble “ (assessor 1) “Focus-bubble “ (assessor 2)	Emotional: Zone

*FAI-G* The Functional Assessment Interview [42], adapted for gambling

**Table 4:** Frequencies of constructs endorsed by total number of participants and specific Pathway subtypes

		Pathway subtypes		
FAI-G section	All *n* = 6	C *n* = 2	E *n* = 2	I *n* = 2
Antecedents that increase gambling	(n phrases per construct)			
Emotion (positive or negative)	6 (50)	2 (14)	2 (18)	2 (18)
Resources (money)	6 (12)	2 (4)	2 (5)	2 (3)
Location	6 (11)	2 (3)	2 (4)	2 (4)
Social stimuli (absence or presence)	6 (10)	2 (3)	2 (2)	2 (5)
Time of the day	5 (12)	2 (6)	2 (4)	1 (2)
Behavior (specific activities)	4 (9)	1 (1)	2 (4)	1 (4)
Specific discriminative stimuli	2 (4)	2 (4)	0 (0)	0 (0)
Gambling losses	2 (3)	1 (2)	1 (1)	0 (0)
Substance use	2 (3)	0 (0)	1 (1)	1 (2)
Antecedents that decrease gambling				
Social stimuli (absence or presence)	6 (12)	2 (3)	2 (5)	2 (4)
Emotion (positive or negative)	5 (15)	2 (8)	2 (4)	1 (3)
Time of the day	5 (9)	2 (4)	2 (4)	1 (1)
Behavior (specific activities)	4 (8)	1 (1)	2 (5)	1 (2)
Location	3 (3)	1 (1)	2 (2)	0 (0)
Experiences when not being able to gamble				
Non-problematic response	4 (12)	2 (5)	0 (0)	2 (7)
Frustrative non-reward	2 (5)	0 (0)	2 (5)	0 (0)
Accompanying responses				
Emotion (positive or negative) "arousal"	3 (7)	1 (1)	1 (1)	1 (5)
Strategies to control gambling				
Avoidance	4 (9)	1 (3)	1 (1)	2 (5)
Monetary based strategies	3 (4)	1 (1)	1 (1)	1 (2)
Social strategies	2 (5)	0 (0)	1 (4)	1 (1)
Strategies to continue gambling				
Antecedent enable (resources)	6 (12)	2 (3)	2 (4)	2 (5)
Antecedent behavior	3 (4)	0 (0)	1 (2)	2 (2)
Consequences of gambling				
Emotional: Positive	6 (31)	2 (12)	2 (12)	2 (7)
Emotional: Zone	6 (17)	2 (4)	2 (9)	2 (4)
Emotional: Avoidance aversives	5 (33)	2 (7)	2 (14)	1 (12)
Tangible: Money	4 (10)	2 (7)	2 (3)	0 (0)
Terminating events of gambling				
Depleted resources (money, physical, time)	6 (8)	2 (2)	2 (4)	2 (2)
Behavior (specific activities)	3 (3)	0 (0)	2 (2)	1 (1)

The table indicates the frequency of participants endorsing the behavioral construct, as well as frequency per gambling Pathways Model subtype [18] according to clinical assessment in this study. The number in parenthesis represents frequency of coded phrases representing the construct (e.g., in total 50 participant phrases were analyzed as Antecedent positive or negative emotions under FAI-G section Antecedents that increase gambling)

FAI-G = The Functional Assessment Interview [42], adapted for gambling

#### Antecedents of gambling behavior

Antecedents refer to events that occur prior to gambling behavior, that may increase or decrease the actual behavior.

All participants reported emotional events that were perceived to increase the likelihood of gambling. Emotional events were coded irrespective of their descriptive value into one theme (i.e., emotion), as the distinction between positive and negative emotional valence was far from clear cut. Thus, emotional antecedents could be described in positive terms, as when participants expressed that they often gambled after “feeling good” or “satisfied in life”. Indeed, all participants expressed that they could experience a positive emotional state of anticipation, excitement or exhilaration prior to gambling. Some, but not all, participants described negatively valued emotional antecedents. All but one participant expressed that negative emotions triggered their gambling, for example feeling “bored”, ”anxious”, “worried”, “stressed”, “sad”, or ”restless”. Others reported pre-gambling rumination, for example thinking that they ought not be gambling, or being displeased with relationships or other areas in their life. Overall, however, few participants expressed that specific gambling-related thoughts triggered their gambling, and when they did, it was often in conjunction with a positive emotional experience. Only two participants described thinking of gambling losses as a trigger for gambling. Emotional events were also reported as antecedents that could decrease the likelihood of gambling. Half of the participants described positive emotions, such as “feeling good”, “happy” or “life going in the right direction”. Two participants described that when being in a negative state, such as feeling “down”, “depressed”, “hopelessness”, or “seeing no opportunities”, they seldom gambled. Thus, emotional events could be considered as a functional theme in understanding conditions that govern gambling, whether positive or negative, and whether they increased or decreased the likelihood of gambling.

Another prominent pattern was that all participants reported that available resources (i.e., access to money) were a critical antecedent condition. For example, participant 3 described a monthly pattern where he gambled using all his salary as soon as the amount was transferred to his bank account. From there on, he lived without money for a couple of weeks feeling pretty good at not gambling and often thinking that he did not want to gamble again. However, as soon as the new salary was transferred to his bank account, he started to gamble online again until the salary was spent, often gambling the whole night long.

Social antecedents were also described by all participants. Social stimuli that were reported to increase gambling were mainly being alone (absence), while decreased gambling was mainly associated with being in contact with others (presence). However, exceptions to gambling alone were noted, for example when friends suggested gambling. Time of day was reported by all but one participant as an antecedent that might increase (mainly evenings) or decrease (mainly daytime and nights) the likelihood of gambling. In the same vein, specific locations were noted by all participants as antecedents that would increase gambling (e.g., “home”, “in my room”, “at public transportations”, or “at work”), but only 3 participants reported locations that were associated with decreased likelihood of gambling (e.g., “outside home” or “outside bedroom”).

A majority of the participants described specific preceding behaviors that either would increase or decrease the likelihood of gambling. Typical activities that would facilitate gambling included for example “browsing gambling Facebook groups”, “ruminating”, or “reading gambling statistics”. Behavior that influenced in the opposing direction typically had the character of competing responses or activities (i.e., behaviors incongruent with gambling). Further, three themes of antecedents were identified with sole functions of increasing gambling. Two participants reported specific discriminative stimuli; that is, events that would clearly signal the availability of reinforcers following gambling behaviors (e.g., gambling commercials). Losses were reported by two persons as antecedents that would increase the likelihood of gambling. Also, use of substances (alcohol and prescribed drugs) was reported by two persons.

#### Experiences when not being able to gamble

Participants were asked to describe their experiences of not being able to gamble. Two main functional themes were identified. One theme identified was frustrative non-reward, for example “frustration” and “irritation” or “can’t focus”. The second theme concerned the more common response which was to describe an essentially non-problematic response, such as “no anxiety or depression”, “I can interrupt gambling”, or “I can focus on other things”.

#### Accompanying responses

The participants were asked to identify physiological responses that would occur regularly when gambling. Three participants described positive or negative emotional arousal-related responses, such as “itchy fingers”, “pumping”,”endorphin-kick”, “fear in the body”, “excitement”, or “itchy body”. The other three did not report any such responses.

#### Strategies to control or continue gambling

Loss of control is a key criterion for GD [3]. The participants were asked to describe their attempts or strategies for controlling their gambling behavior. The main functional theme described concerned avoidance-based strategies, such as not owning a smartphone or a bank card reader, handing over control over their economy to significant others, blocking gambling accounts or credit cards, or extracting money in cash to prevent themselves from gambling.

*“During a one-year period I handed over my finances to my brother. I also got help with budget and… making calls and so forth. I didn’t gamble for...surely one and a half years. Everything was great, but then I got it back [control over my finances]. After that I started to gamble again pretty fast.” (Participant 3)*

The other strategies for controlling gambling were labeled either social strategies, for example scheduling non-gambling activities with friends, or telling their friends they had gambling problems and prohibiting them from lending them money; or monetary-based strategies, such as ceasing to borrow money for gambling, depositing only small sums in gambling accounts, or saving money to cover other minimum living expenses.

In contrast, gambling also involves using a variety of strategies that serve the opposite function: deliberate planning that enables or facilitates gambling. These responses were categorized either under the heading of enabling or securing resources, or as different kinds of planned and deliberate behaviors, for example taking out loans to gamble or cover other expenses, waiting for salary, selling possessions, lying or gambling to win, or to win back money.

#### Consequences of gambling

Participants were asked to describe the experiences and events that would either accompany or occur subsequent to their gambling behavior, in order to identify the possible reinforcing properties of gambling and its contextual factors. These were identified as either different emotional properties or tangible reinforcers (i.e., money).

*“There were different stages. (...) First it felt like a big development for me, that I had found something (...) it was a feeling of great… a good feeling. I was happy with myself and felt I was going somewhere. Then, when the winnings were replaced with losses, and the bettings became wilder (...) I remembered my first feeling, that I had won (...) In the next step I betted more aggressively, to, sort of like, catch up to what I potentially thought I should have won (...).* It became a straitjacket pretty fast when the losses mounted up and I started to chase them.” (Participant 6)

Overall, participants described that they experienced a range of emotional states while they gambled; these we categorized as positive (Emotional positive). Common descriptions were “excitement”, “kicks”, “euphoria”, “satisfaction”, or feelings of “being in control”, “being on the right path”, “invincibility”, or “growing ego”. Another functional theme was that gambling was described as serving a function to avoid aversive emotional experiences, for example “getting a break”, “escaping reality”, avoiding “responsibility”, “social interaction”, “boredom”, or “bad conscience”, or avoiding hard thoughts of “debts”, “betrayal against family” or “social failures”. All but one participant described that anxiety was fulfilling different functions in their gambling experiences. By gambling, participants avoided symptoms of anxiety, for example post-mortem ruminations on social situations, or post-traumatic memories. Interestingly, anxiety was also described as a part of the gambling activity in itself. Participants described that gambling “is a mixture between excitement and anxiety”, and “relieves anxiety in the short term, but increases it in the long term”, or “relieves gambling-related anxiety in the short term”, but it was also described as “a relief when the money's gone”, and there being a point where “it gets calm in the head”.

*“Nowadays I see my gambling as a form of deliberate self-harm. Uumm*… Because now I don’t gamble to… I know that I can win a lot of money. But if I win a lot of money, I will use it to gamble anyway. I rarely gamble to win, I only gamble to shield myself from reality.“ (Participant 4)

A third functional theme, “the zone”, involved participants’ experience of a state of selective attention, or focus, while they gambled. This state was described mainly in positive terms as “focus”, “being able to concentrate”, “entering a bubble”, or “all thoughts on gambling”, and was often associated with a feeling of escaping reality (sometimes also avoiding aversive thoughts or feelings), tunnel vision, lost perception of time, as well as continuing to gamble until all money were gone. For example, Participant 3 expressed that:

*“I get totally stuck. I know situations where I gambled for, what I perceived as half an hour, fifteen minutes, twenty minutes. But instead… well, one and a half hours* had gone by. What? I sort of like lose perception of time (...) when I win and… perceive that it goes well, and later, when you click and click, then... Well, out of money. But it went well fifteen minutes ago (...) Often when I gamble, I feel best.”

As expected, money was identified as a tangible reinforcer for gambling behavior. However, while all participants described emotional consequences, only four of them explicitly reported money as an important consequence. Overall, participants described that winning was associated with “a great feeling”, “a kick” or “euphoria”, but also that winnings resulted in “feelings of unreality”, and that the money they won lost its value and became “just numbers on the account”. Two participants described that they “chased wins”, “chased absent wins”, or “demanded absent wins” when they gambled. Participant 1 expressed that he knew he could not win money by gambling, but that these thoughts were “blocked in the brain somehow” while he gambled. Overall, participants described that losses during gambling were associated with feelings of “anger”, “frustration”, “anxiety” and “a lust for revenge”. Half of the participants described that they continued to gamble to “win back money” or “chase losses”.

#### Terminating events of gambling behavior

The participants were asked to identify circumstances that would terminate a period of gambling behavior. We identified two broad functional themes. The first was depleted resources, which included running out of money. All participants reported this. But it could also be physical or temporal resources, such as continuing to gamble until becoming exhausted and falling asleep or running out of time.

The second theme mainly consisted of different behaviors that served the function of terminating gambling. For example, participant 5 described that gambling sessions usually ended according to her pre-decided plan. Notably, only participant 6 described a specific time as a terminating event for gambling. This participant was the only one who gambled on the stock market (day trading) which was not accessible around the clock, as were other gambling types played online by the remaining participants.

#### Behaviors with functions similar to gambling

Finally, participants were asked to report other behaviors that they had engaged in, that resembled the experience of gambling. Four participants described various behaviors and activities, i.e., computer games, other games, sex, deliberate self-harm, and work tasks. However, we decided that these behaviors were too disparate to constitute a functional theme and they were therefore excluded from the thematic analysis.

#### Pathways Model subtypes

When comparing the clinically assessed Pathway subtypes, few clear differences in the functional properties of gambling were found. Instead, a more general gambling pattern was identified which seemed to include all participants, irrespective of subtype categorization. However, two differences were noted. First, the only participants who described frustrative non-reward responses were the two participants clinically assessed as emotionally vulnerable. Secondly, somewhat surprisingly, three participants, assessed as emotionally vulnerable and impulsive Pathway subtypes, described behaviors that enabled them to stop gambling. For example, participant 2, assessed as an impulsive gambler, described that he could stop gambling while still having money in his account if:

*“(...) someone wants to go out and do something fun. If something else is happening, not just going out to drink beers. To go bowling, we go and do this, do you want to come and bathe* in the sauna or go swimming. To do things.”

## Discussion

This study used a functional assessment interview and self-report measures to identify clinically relevant behaviors and formulate hypotheses on the maintenance of GD on an individual level, as a preparatory step for guiding clinical interventions. The Pathways model subtypes [18] were used to obtain a diverse sample of participants with GD. The study was driven by an overarching interest in clarifying the functional aspects of gambling, from a subjective participant perspective.

When investigating the context of gambling behavior, a striking feature was that study participants often reported commonplace antecedents, such as being alone, time of the day (e.g., evenings), and being at home. The most prominent antecedent reported, indeed, was access to money. This suggests that gambling could be viewed as a secluded activity, mainly triggered by access to money. For our participants, who mainly gambled online, it was possible to gamble everywhere and at any time, with the only hindrance that money was not equally available. Depleted financial resources were, consequently, described as the main terminating event of gambling by the participants. Some also described physical exhaustion or running out of time. Loss of control is often regarded as a defining feature of GD [3, 18]. Our results indicates that it could be more complicated. All participants in this study described that they had used stimulus control strategies in order to refrain from gambling. While stimulus control strategies are endorsed among recovered gamblers [33], and typically employed as a first intervention in many treatment protocols [27], they are essentially avoidance-based strategies. One drawback with such strategies may be that the person fails to learn control of behavior in the presence of the antecedents that tend to result in gambling behavior.

Gambling is often described as an escape from negative emotions and aversive experiences [3, 18]. Our results indeed indicated emotional antecedents for gambling. However, the link to negative emotions was not exclusive. Some participants described that positive emotions preceded their gambling, and others that negative emotions did so. Conversely, some participants expressed that positive emotions decreased the possibility for them to gamble, and others that negative ones did so. However, it should be noted that all participants expressed that they experienced an emotional state of expectancy prior to gambling. Gambling-related physiological arousal and subjective excitement is consistent with the theoretical Pathways Model [18], and has been examined in several experimental studies e.g., [50–52]). For example, Rockloff and Greer [53] concluded that high arousal can increase subsequent gambling behavior among at-risk players, as long as the arousal is not perceived as a negative emotion. Thus, future etiological and treatment models may consider affective antecedents regardless of valence.

The participants’ descriptions of the relationship between gambling and winning or losing money were not unanimous. While all participants but one scored above clinical cut-off at “Winning and Chasing” on the CAMH-IGS, only four of them explicitly reported money as an important consequence of gambling. Two participants described that they chased wins, and three participants that they continued to gamble to win back money they lost. The gambling activity itself was also described in relation to emotional events, where placing a bet was associated with excitement, winning with euphoria and a kick, and losing with anxiety and a lust for revenge; findings that are in line with a functional magnetic resonance imaging study by Campbell-Meiklejohn et al. [54]. Chasing, in particular chasing losses, has been proposed as a key symptom of GD [55], although experimental studies investigating this phenomenon seem rare [29]. Our results suggest that tangible reinforcers; i.e., money, might be important for gambling behavior, but probably do not account for the whole clinical picture of GD.

A more striking feature in the participants’ narratives was that they all reported a positive state of selective attention, or focus, while they gambled. While this “zone” typically is not part of existing gambling treatment protocols [27], nor of the Pathways Model [18], it is not a novel finding. As previously noted, Schüll [31], downplayed winning money as a motivating factor, and instead described the slot machine as a “zone”, where events occurring outside the gambling experience become less relevant to gamblers, as they grow completely absorbed by the game. Similarly, Dixon et al. [56] coined the expression “dark flow”, a flow-like state which has been investigated in experimental studies and found to be associated with multiline slot gambling and GD [56, 57]. Findings from the present study are in line with the presumption that this state might be an important reinforcer for gambling behavior.

Inherent in the idea of an addiction lies the idea of craving, coupled with experiencing abstinence when access to the drug is hindered. In parallel with this, abstinence is a diagnostic criterion of GD [3]. Somewhat surprisingly, two participants in this study reported not being able to gamble as entirely non-problematic, i.e., other activities enabled them to stop gambling fairly easily despite having access to money. They experienced no negative symptoms, such as anxiety, depression or concentration problems. It should be noted that both participants were assessed as impulsive gamblers according to the Pathways Model [18], which may indicate a unique feature of this theme.

This study had several strengths. Gambling has been investigated in previous qualitative studies, but not from a clinical perspective. As previously noted, this is a sensible strategy, as treatment interventions ideally should emanate from ideographic models. Interviewing “sufferers” is often conducted to identify hypotheses of maintenance for problem behaviors, when developing novel CBT (34,35, personal communication Edna Foa). These qualitative and clinical based assessment procedures are, however, rarely published as formal systematic studies. The current paper is thus an important exception in the clinical treatment literature. This study used theory-based clinical sampling. Participants from a CBT study were purposely selected by a clinical psychologist. This ensured both richness of data and that participants were familiar with the behavioral constructs in the FAI-G interview and self-report measures.

Limitations of the study included the lack of validation of results and conclusions by reporting them back to the participants. Also, we did not use a predefined procedure to assess whether saturation was reached. However, we found that code saturation was achieved following recruitment of six participants, in line with findings by Henning et al. [58], who have studied the saturation process and found that over 80% of coding can be expected after six interviews. The first interviews generated a rich range of coding, and for the purposes of this study six participant sufficed. The use of a semi-structured interview format, based upon a predefined theoretical framework, delimited possible conclusions in the thematic analysis, and created difficulties, for example, in differentiating themes from constructs. Also, theoretical (i.e., functional, and behavioral) terms were used throughout the data collection, which might have hindered the participants’ understanding of the questions being asked. However, as the participants had undergone a recent cognitive behavioral treatment study [37], where individual clinical behavioral analyses were continuously performed, they were familiar with the theoretical constructs employed in the current study. Overall, the study was a preparatory step for developing a CBT treatment protocol, so other methodological approaches would probably have been of less clinical relevance.

With regards to the functional aspects of gambling, this study has merits from a heuristic perspective since it identified several potential processes which might be clinically relevant for GD, but typically have not been part of gambling treatment protocols (e.g., [27]). In terms of clinical implications, a treatment model and an internet-based cognitive behavioral protocol was developed, based on the results of the study. The treatment was disseminated into routine addiction care and is currently being evaluated in a feasibility study (see 28 for a study protocol). The interviews and results of the current study were completed before the treatment development and feasibility study was initiated.

In sum, the aim of the current study was to assess the subjective functions of gambling, within a diverse sample of participants with GD, ultimately with the goal of informing treatment development. The considerations could be important to address in future CBT models and treatment protocols for GD. First, access to money might be a critical antecedent for GD, and we question the use of avoidance-based control strategies in treatment if the objective is to achieve long-term control over gambling behavior. Secondly, treatment needs to address both negative and positive antecedent emotions for gambling behavior (e.g., anticipation), and not only negatively reinforced gambling behavior. Third, the gambling activity in itself seems to include emotional functions. In particular, an absorbing experience of selective attention during gambling might be an important reinforcer, and should accordingly be addressed in CBT protocols. Finally, gamblers in the impulsive subtype did not report experiencing abstinence symptoms when not being able to gamble, despite presence of critical antecedents, such as access to money. Future clinical studies could investigate this phenomenon further, using targeted interventions, such as behavior replacement.

## Conclusions

- Subjective functions of gambling behavior were identified among a sample of participants with gambling disorder, as a means to guide new developments in cognitive behavioral interventions.
- Access to money might be a critical antecedent for gambling and should be addressed using non-avoidance interventions.
- Treatment should address positive and negative emotions both as potential antecedents and functions of gambling behavior.
- Anticipation, selective attention, and chasing might be important reinforcers for gambling.

## Supplementary Information

**Additional file 1. **Functional assessment interview for gambling (FAI-G).

## Data Availability

The data that support the findings of this study are available on request from the corresponding author. The data are not publicly available due to privacy or ethical restrictions.

## Abbreviations

AHB: Anne H Berman, last author
APA: The American Psychological Association
CBT: Cognitive Behavioral Treatment
CAMH-IGS: The Centre for Addiction and Mental Health Inventory of Gambling Situations
COREQ: The Consolidated Criteria for Reporting Qualitative Research
DSM-5: The Diagnostic and Statistical Manual of Mental Disorders, 5th edition.
FAI-G: The Functional Assessment Interview
GD: Gambling Disorder
GFA: The Gambling Functional Assessment
GPQ: The Gambling Pathway Questionnaire
JB: Johan Bjureberg, third author
JR: Jonas Ramnerö, second author
PGSI: The Problem Gambling Severity Index
MINI-7: The Structured Clinical Interview for Gambling Disorder, version 7
OM: Olof Molander, first author
REGAPS: Responding to and Reducing Gambling Problems Studies
SCI-GD: The Structured Clinical Interview for Gambling Disorder
