# Supplementary material for: What to target in cognitive behavioral treatment for gambling disorder—A qualitative study of clinically relevant behaviors
Source: BMC Psychiatry. 2022 Jul 28;22:510. doi: 10.1186/s12888-022-04152-2 (PMC9331573; doi:10.1186/s12888-022-04152-2)
Supplement: Supplementary file 1 — Additional file 1. Functional assessment interview for gambling (FAI-G). [file 12888_2022_4152_MOESM1_ESM.pdf]

FUNCTIONAL ASSESSMENT INTERVIEW FOR GAMBLING (FAI-G)
Research interview

Name:			
Age:		Gender:	
Date:		Interviewer:	
Location:			

Adapted for Gambling Disorder by Olof Molander, Jonas Ramnerö and Viktor Månsson, Karolinska Institutet 2017; from: O'Neill, R. E., Horner, R, H., Albino, R. W., Sprague, J. R. Storey, K. & Newton, J. S. (1997). *Functional Assessment and Program Development for problem Behavior; A practical handbook.* Pacific Grove: An International Thompson Publishing Company, Brooks/Cole Publishing Company.

1. Descriptive questions

1.1. How often do you gamble?	
1.2 For how long?	
1.3 For how much money?	
1.4 Do your stakes increase gradually, or do you gamble for about the same amount?	

1.5 What type(s) do you usually gamble?

Casino online	Sports online	Poker online	Vegas	Lotteries	Bingo
Casino venue	Sports venue	Poker club	Number games	Horses	Other types

1.6 What does gambling mean to you (describe the experience)? Do you like to gamble? If so, what do you appreciate about it? Have you experienced that you sometimes want to gamble, even if you do not like it?

1.7. When did you gamble for the first time?

1.8 When did you notice that you had gambling-related problems?

1.9 Do you gamble mostly alone, or with others?

1.10. Have others commented on your gambling? How? Has your gambling created problems in your relationships in any way? Have you ever lied about your gambling, e.g., the scope of it, or sums betted?

1.11 Do you use any medications?

1.12 Do you use alcohol or drugs? Do you use alcohol/drugs when you gamble?

1.13 Would you say that, in addition to gambling, you are a person who usually exposes yourself to risky situations? How? Which situations? Please describe.

1.14 Would you say that you are a person who can handle "boring" situations? How? Please describe.

2. Antecedents

2.1 Where do you usually gamble?

2.2 Do you usually **think** of something just *before* you gamble?

2.3 Do you usually **feel** something (how it feels in your body) just *before* you gamble?

2.4 Do you usually **do** something just *before* you gamble?

2.5 What happens when you are **not allowed/can't** gamble?

2.6 Are there specific sounds, lights, flavors, emotions or things that are directly related to gambling for you? Which? In what contexts are you most likely to encounter these?

3. During gambling

3.1 Describe your experience while gambling (during the gambling)?

3.2 Are there any special thoughts and/or feelings that are associated with your gambling?

3.3 How do you react to losses?

3.4 What usually makes you want to stop the gambling?

3.5 When do you usually stop the gambling?

3.6 Do you have any special strategies to be able to continue to gamble another day? (e.g., get more money, keep gambling a secret…)

6. Antecedents

	**Most likely**	**Least likely**
Feelings (emotions) that most/ least likely lead to gambling?		
When (time of day) most/least likely gambling occurs?		
Where does most/least likely gambling occur?		
With whom does gambling most/ least likely arise?		
What *other behaviors* most/least likely lead to gambling?		

7. Function and maintenance

Positive reinforcement<br>"Is there anything you get out of gambling?"	*Negative reinforcement*<br>"Is there anything you avoid by gambling?"

8. Other behaviors

Other behaviors<br>"Are there other similar behaviors that work in the same way as gambling for you?”<br><br>E.g., alcohol, drugs, shopping, sex<br>(rank by how similar 1-10)	”Describe why”

9. Summary assessment (interviewer)

Circumstances that *increase* the probability of gambling	
Circumstances that *reduce* the probability of gambling	
Antecedents/triggers for gambling	
Verbally described physiological components	
Own strategies to control gambling	
The described function of gambling	
Other similar behaviors	
Suggestions for interventions	
